# Maternal per- and polyfluoroalkyl substances exposure and the co-occurrence of gestational diabetes mellitus and preeclampsia/eclampsia: a nested case–control study

**DOI:** 10.3389/fpubh.2026.1929511

**Published:** 2026-09-03

**Authors:** Nana Li, Lu Li, Ying Deng, Meixian Wang, Yuting Li, Zhen Liu, Hong Kang, Jun Zhu, Ping Yu, Yanping Wang

**Affiliations:** 1 National Center for Birth Defect Monitoring, West China Second University Hospital, Sichuan University, Chengdu, Sichuan, China; 2 Key Laboratory of Birth Defects and Related Diseases of Women and Children (Sichuan University), Ministry of Education, Chengdu, Sichuan, China

**Keywords:** co-occurrence, eclampsia, gestational diabetes mellitus, per- and polyfluoroalkyl substances, preeclampsia

## Abstract

**Objective:**

Few studies have investigated the relationship between maternal exposure to per- and polyfluoroalkyl substances (PFAS) and the co-occurrence of gestational diabetes mellitus (GDM) and hypertensive disorders of pregnancy, particularly preeclampsia/eclampsia (PE/E). The present study aimed to explore whether PFAS exposure is associated with the co-occurrence of GDM and PE/E.

**Methods:**

This study enrolled 333 pregnant women diagnosed with both GDM and PE/E, and 360 healthy controls. First-trimester plasma concentrations of 18 PFAS were determined by ultra-high performance liquid chromatography-tandem triple quadrupole mass spectrometry (UPLC-MS/MS). The associations of individual and combined maternal PFAS exposure with the odds of co-occurrence were assessed by restricted cubic spline (RCS) analysis, multivariable logistic regression, and Bayesian kernel machine regression (BKMR).

**Results:**

The concentrations of perfluoroheptanoic acid (PFHpA), perfluorobutanesulfonic acid (PFBS), and perfluorotridecanoic acid (PFTrDA) differed significantly between the two groups; however, after false discovery rate (FDR) correction, only PFHpA and PFTrDA remained significantly different. RCS analysis showed significant associations of PFHpA and PFBS with the co-occurrence (*P*_overall_ = 0.026 and 0.014, respectively), without nonlinearity (*P*_non-linear_ = 0.619 and 0.206, respectively); However, the overall associations were no longer significant after FDR correction. In multivariable logistic regression models, after FDR correction, each log10-unit increment in PFHpA and PFBS was significantly associated with increased odds of the co-occurrence, with adjusted odds ratios (aORs) of 2.439 (95% CI: 1.269–4.689) and 1.688 (95% CI: 1.161–2.453), respectively. For perfluoroheptane sulfonic acid (PFHpS), a positive association was also observed (aOR = 1.712, 95% CI: 1.051–2.788); however, the association was not significant after FDR correction. BKMR analysis revealed a positive association between PFAS mixture exposure and the co-occurrence, with the association strengthening progressively at higher mixture concentrations. Among the individual PFAS, PFHpA, PFBS and PFHpS exhibited positive trends, whereas PFTrDA displayed inverse trends.

**Conclusion:**

PFHpA, PFBS, and the 10 PFAS mixture were all positively associated with increased odds of co-occurrence of GDM and PE/E.

## 1 Introduction

Gestational diabetes mellitus (GDM) and hypertensive disorders of pregnancy (HDP) are widely recognized as common pregnancy-related complications. GDM is typically defined as glucose intolerance arising or first recognized during pregnancy (1). Globally, approximately 14% of pregnant women are affected by GDM (2), with a prevalence of 14.8% in mainland China (3). HDP encompass a variety of conditions marked by elevated blood pressure during gestation, including chronic hypertension, gestational hypertension, and preeclampsia or eclampsia (PE/E) (4), affecting approximately 5 to 10% of pregnant women worldwide (5), and an estimated 7.30% in China (6). GDM and HDP are linked to a spectrum of unfavorable perinatal outcomes, such as fetal growth disturbances (intrauterine growth restriction and macrosomia), low birth weight, and preterm birth, alongside long-term health risks (7). Furthermore, HDP are recognized as major contributors to global maternal and fetal morbidity and mortality (8). Collectively, GDM and HDP impose a substantial health and economic burden on pregnant individuals and their offspring.

Notably, a close clinical association exists between GDM and HDP. Pregnant women with GDM are at significantly higher risk for developing hypertension and preeclampsia (9), and glucose-lowering treatment for GDM has been shown to reduce the subsequent risk of HDP (10, 11). Multiple studies have further identified GDM as an independent risk factor for preeclampsia (12). The comorbidity of GDM and HDP produces a synergistic effect, further elevating risks for both mothers and newborns (13). For instance, compared with either condition alone, the co-occurrence of HDP and GDM is significantly associated with elevated risk of both small-for-gestational-age (SGA) and large-for-gestational-age (LGA), exhibiting a “bidirectional risk” pattern toward both extremes of fetal growth (14). Another study has reported that GDM and HDP comorbidity is associated with higher neonatal morbidity risk than either condition alone (15). While advanced maternal age, overweight or obesity, nutrition are recognized as common risk factors for GDM and HDP, the risk factors specifically associated with their co-occurrence remain largely unexplored (7).

Per- and polyfluoroalkyl substances (PFAS) are a large class of synthetic chemicals characterized by a fluorinated aliphatic carbon backbone (16). Their unique lipophobic, hydrophobic, and thermally stable properties have driven widespread industrial and consumer applications, including aqueous film-forming foam, textile manufacturing, metal-plating, cosmetics, food packaging, and nonstick cookware (16, 17). Due to their strong carbon-fluorine bonds, PFAS are highly resistant to degradation, resulting in environmental persistence, bioaccumulation, and widespread human exposure through contaminated drinking water, fishery products, indoor air, and consumer goods (18, 19). Consequently, PFAS are detectable in various human biological specimens, including plasma, serum, cerebrospinal fluid, milk, and urine (20).

Pregnant women are considered a particularly vulnerable population to environmental chemicals exposure due to their altered physiological state (21). Accumulating evidence suggests that PFAS may interfere with maternal glucose metabolism and blood pressure regulation through multiple mechanisms, including impairment of placental function and angiogenesis, and induction of oxidative stress and systemic inflammation (22–25). These pathways collectively provide plausible biological links between PFAS exposure and the development of GDM and HDP.

However, epidemiological evidence regarding the association between PFAS exposure and pregnancy complications remains inconsistent. For GDM, some studies have reported positive associations with PFAS exposure (26–28), while others have suggested an inverse (29) or null (30, 31) associations. Similarly, for HDP, findings have been heterogeneous, with positive (32–36), null (37, 38), or inverse (39, 40) associations reported across different study populations and PFAS congeners. These discrepancies may be attributable to differences in study design, sample size, population characteristics, biological sample types, and adjustment for confounding factors. Notably, no epidemiological study to date has investigated whether PFAS exposure is associated with the co-occurrence of GDM and HDP, a clinically relevant comorbidity that may have distinct etiological underpinnings from either condition alone.

To fill this research gap, we conducted a nested case–control study based on the maternal drug exposure birth cohort (DEBC) in China. We quantified 18 PFAS compounds in first-trimester maternal plasma to explore their individual and combined associations with the co-occurrence of GDM and PE/E.

## 2 Materials and methods

### 2.1 Study population

This nested case–control study was derived from the DEBC, which prospectively enrolled pregnant women from August 2018 through December 2021 (41). Briefly, inclusion criteria for pregnant participants were as follows: (1) gestational age below 14 weeks at their initial prenatal appointment, (2) intended to establish medical records and deliver at the enrolling hospital, and (3) understood the study objectives and provided written informed consent. Exclusion criteria comprised mental health disorders precluding questionnaire completion, intrauterine fetal death at the initial prenatal ultrasound, and HIV-positive. Following enrollment, participants completed trimester-specific questionnaires and provided 4 mL EDTA-anticoagulated blood during the first and second trimesters. After centrifugation, the plasma was aliquoted and preserved at −80 °C for subsequent assays.

This nested case–control study comprised 693 participants, including 333 cases with GDM complicated by PE/E, and 360 normal controls without any pregnancy complications. To be assigned to the case group, participants needed to satisfy three requirements: (1) singleton pregnancy, (2) diagnosis of both GDM and PE/E, and (3) availability of first-trimester plasma samples. Controls were selected based on the following criteria: (1) singleton pregnancy, (2) absence of diabetes, hypertension, or any other gestational complications, (3) availability of first-trimester plasma samples, and (4) enrollment on the same day and at the same hospital as the matched case participants. Data on family history of hypertension and diabetes among first-degree relatives (parents and siblings) were collected via questionnaire. To mitigate potential genetic confounding, any pregnant woman with a positive family history of either condition was excluded from the study. Supplementary Figure S1 displays the flowchart for participant enrollment and exclusion.

### 2.2 Definition of GDM, preeclampsia and eclampsia

All participants underwent standardized 75 g oral glucose tolerance (OGTT) test from 24 to 28 gestational weeks to screen for GDM, with diagnostic standards strictly referencing the International Association of Diabetes and Pregnancy Study Groups (IADPSG) guidelines (42). A diagnosis was established if any of the following venous plasma glucose thresholds was reached: fasting value ≥ 5.1 mmol/L, 1-h value ≥10.0 mmol/L, or 2-h value ≥ 8.5 mmol/L.

Preeclampsia and eclampsia were diagnosed according to the clinical practice guideline in China (43). Preeclampsia was defined as hypertension that first appeared after 20 gestational weeks (systolic blood pressure (SBP) ≥ 140 mmHg and/or diastolic blood pressure (DBP) ≥ 90 mmHg), in combination with proteinuria and/or maternal organ dysfunction and/or uteroplacental dysfunction. Proteinuria is defined as 24-h urinary protein excretion ≥ 0.3 g, or urinary protein to creatinine ratio ≥ 0.3, or random urine protein ≥ 1+. Eclampsia was defined as seizure onset occurring in preeclamptic women, with other causes of seizures rigorously excluded.

The information on GDM, preeclampsia and eclampsia was obtained from medical records.

### 2.3 Quantification of per- and polyfluoroalkyl substances

Maternal first-trimester plasma samples were analyzed using ultra-high performance liquid chromatography-tandem triple quadrupole mass spectrometry (UPLC-MS/MS), performed on an ACQUITY UPLC I-Class system (Waters, United States) coupled with a triple quadrupole mass spectrometer (Xevo TQ-XS, Waters, United States), equipped with an ACQUITY UPLC BEH C18 VanGuard guard column (Waters, United States). The analyses were conducted at the West China School of Public Health, Sichuan University. The detailed method for measuring PFAS has been published previously (44).

The target PFAS included perfluorohexane sulfonic acid (PFHxS), perfluoroheptane sulfonic acid (PFHpS), perfluorooctane sulfonic acid (PFOS), perfluorodecane sulfonic acid (PFDS), perfluoroheptanoic acid (PFHpA), perfluorooctanoic acid (PFOA), perfluorononanoic acid (PFNA), perfluorodecanoic acid (PFDA), perfluoroundecanoic acid (PFUnDA), perfluorododecanoic acid (PFDoA), perfluorotridecanoic acid (PFTrDA), perfluorotetradecanoic acid (PFTeDA), perfluorobutanoic acid (PFBA), perfluoropentanoic acid (PFPeA), perfluorobutanesulfonic acid (PFBS), perfluorohexanoic acid (PFHxA), perfluorohexadecanoic acid (PFHxDA), and perfluorooctadecanoic acid (PFODA). All detected concentrations that were below the limit of detection (LOD) were subsequently assigned a value of LOD/ $\sqrt{2\;}\;$ (45).

### 2.4 Covariates

The covariates were identified by referencing previous studies and a directed acyclic graph (Supplementary Figure S2) and were subsequently adjusted for in regression models. These encompassed maternal age, pre-pregnancy body mass index (ppBMI), maternal ethnicity, maternal education level, method of conception, parental tobacco smoke exposure, maternal alcohol consumption, gravidity, parity, enrollment season. Detailed definitions and categorizations of these variables are provided in Supplementary Table S1.

### 2.5 Statistical analyses

There were no missing data for any of the analytical variables, as plasma sample availability and complete covariate information were inclusion criteria. No imputation methods were applied. Participants’ demographic characteristics were described as median with interquartile ranges (IQR) for continuous variables due to skewed distributions, and number (%) for categorical variables, with between-group comparisons made using the Mann–Whitney *U* test and the Chi-squared test, respectively.

The PFAS concentrations were presented as medians with interquartile ranges (IQR) due to skewed distributions. Intergroup differences were evaluated using the Mann–Whitney *U* test. To reduce potential bias arising from frequent undetectable values, the analysis was confined to PFASs with a detection frequency of ≥70%. The pairwise correlation across raw PFASs concentrations was assessed by Spearman rank correlation analysis. A log10-transformation was applied to the raw PFAS concentrations for subsequent analysis.

Restricted cubic spline (RCS) models were fitted to identify nonlinear associations between PFAS concentrations and the odds of co-occurrence of GDM and PE/E. Knots were placed at the 10th, 50th, and 90th percentiles of PFAS levels, with the 10th percentile serving as the reference point. The Wald *χ*^2^ test was employed to examine the overall association and potential nonlinear relationships. Additionally, multivariable logistic regression models were employed to examine the associations of continuous individual PFAS exposures with the co-occurrence of GDM and PE/E, with results presented as odds ratios (ORs) and 95% confidence intervals (CIs) per log10-unit increase in each PFAS. All analyses were adjusted for the covariates described above.

Bayesian kernel machine regression (BKMR) model was employed to investigate the associations of individual and combined maternal PFAS exposure with the odds of co-occurrence of GDM and PE/E. The models were adjusted for the covariates described above. The combined associations of PFAS was displayed by evaluating the expected change in the odds of co-occurrence of GDM and PE/E, when 10 PFAS were fixed at particular percentiles (i.e., from 25th to 75th percentile, with an interval of 5 percentile) compared with the reference odds when all 10 PFAS were in their 50th percentile. The exposure–response function for each PFAS was presented by fixing all other nine PFAS at their 50th percentile. The bivariate exposure-response function for each PFAS was depicted conditional on the secondary PFAS being fixed at the 10th, 50th, or 90th percentile, with the other eight at their 50th percentile. Individual posterior inclusion probabilities (PIPs) were calculated to identify the key contributory PFAS.

To further evaluate whether the extreme imbalance in assisted reproductive technology distribution between cases and controls affected the stability of our estimates, sensitivity analyses were conducted restricted to naturally conceived participants. Additionally, subgroup analyses stratified by maternal age (categorized as <35 years vs. ≥35 years) were conducted.

All statistical analyses were carried out using SPSS (version 16.0, SPSS Inc., IBM, Chicago, United States) and R (version 4.4.3, 46). The RCS and BKMR models were performed using the “rms” and “bkmr” packages in R, respectively. False discovery rate (FDR) correction using the Benjamini-Hochberg method was applied to adjust for multiple comparisons. Statistical significance was set at a two-tailed *p*-value of less than 0.05.

## 3 Results

### 3.1 Demographic characteristics of the participants

Demographic characteristics of all participants are displayed in Table 1. Significant intergroup differences were observed in pre-pregnancy BMI, method of conception, and parity. Specifically, the case group showed elevated median pre-pregnancy BMI (23.44 [IQR, 20.91–25.86] vs. 20.49 [IQR, 19.08–22.27]), higher proportion of medically assisted reproduction (17.07% vs. 0.28%) and nulliparous women (67.07% vs. 53.31%). In contrast, the two groups did not differ significantly in maternal age, ethnicity, educational level, parental tobacco smoke exposure, maternal alcohol consumption, gravidity, or enrollment season.

**Table 1:** Descriptive characteristics of the participants.

Characteristic	Controls (*n* = 360)	GDM and PE/E (*n* = 333)	*p*-values
Maternal age [years]			0.467
Median (IQR)	31.28(28.25,35.44)	31.82(28.71,35.72)	
Pre-pregnancy BMI [kg/m^2^]			<0.001
Median (IQR)	20.49(19.08,22.27)	23.44(20.91,25.86)	
Maternal ethnicity [*n* (%)]			0.513
Han	325(90.28)	295(88.59)	
Others	35(9.72)	38(11.41)	
Maternal education level [*n* (%)]			0.333
Junior high or lower	48(13.33)	57(17.12)	
High school	58(16.11)	56(16.82)	
College or higher	254(70.56)	220(66.07)	
Method of conception [*n* (%)]			<0.001
Natural conception	359(99.72)	276(82.88)	
Medically assisted reproduction	1(0.28)	57(17.12)	
Parental tobacco smoke exposure [*n* (%)]			0.143
No	180(50)	148(44.44)	
Yes	180(50)	185(55.56)	
Maternal alcohol consumption [*n* (%)]			0.874
No	288(80)	268(80.48)	
Yes	72(20)	65(19.52)	
Gravidity [*n* (%)]			0.237
Primigravida	126(35)	131(39.34)	
Multigravida	234(65)	202(60.66)	
Parity [*n* (%)]			<0.001
Nulliparous	192(53.33)	224(67.27)	
Parous	168(46.67)	109(32.73)	
Enrollment season [*n* (%)]			0.227
Spring	108(30.00)	82(24.62)	
Summer	120(33.33)	106(31.83)	
Autumn	70(19.44)	82(24.62)	
Winter	62(17.22)	63(18.92)	

GDM, gestational diabetes mellitus; PE/E, preeclampsia/eclampsia; IQR, interquartile ranges. *P*-values were derived from the Mann–Whitney *U* test for continuous variables and Chi-square test for categorical variables.

### 3.2 Maternal plasma PFAS levels and their correlations

Maternal plasma PFAS levels between the two groups are summarized in Table 2. Ten of the 18 PFAS (PFHpA, PFOA, PFNA, PFDA, PFUdA, PFTrDA, PFBS, PFHxS, PFHpS and PFOS) were detectable in at least 70% of maternal plasma samples, and was included in the subsequent analysis. Of these, PFOA was the dominant compound detected, with a median concentration of 4.658 ng/mL, followed by PFOS at 3.329 ng/mL. The concentrations of other homologs were ordered as PFNA > PFUnDA > PFDA > PFTrDA > PFHxS > PFHpS > PFBS > PFHpA. Compared with the control group, PFHpA and PFBS concentrations exhibited a significant elevation in the co-occurrence group, while PFTrDA showed an inverse trend. However, after FDR correction, the difference in PFBS concentrations no longer reached statistical significance (FDR *p* > 0.05).

**Table 2:** First trimester maternal plasma PFASs levels (ng/mL) in the study population.

PFASs	LOD	>LOD	Median (IQR)			*P*-values	FDR *P-*values
	(ng/mL)	*n* (%)	Total participants (*n* = 693)	Controls (*n* = 360)	GDM and PE/E (*n* = 333)		
PFOA	0.0022	691(99.71)	4.648(2.791,7.786)	4.592(2.8,7.345)	4.667(2.763,8.201)	0.687	0.858
PFOS	0.0058	691(99.71)	3.308(2.029,5.763)	3.131(1.963,5.808)	3.408(2.083,5.69)	0.865	0.865
PFNA	0.0016	691(99.71)	0.863(0.562,1.364)	0.891(0.561,1.375)	0.858(0.563,1.327)	0.787	0.865
PFHxS	0.005	691(99.71)	0.314(0.181,0.63)	0.311(0.169,0.594)	0.314(0.189,0.653)	0.339	0.678
PFHpA	0.0016	690(99.57)	0.037(0.028,0.058)	0.036(0.027,0.053)	0.039(0.029,0.064)	0.008	0.04
PFHpS	0.006	688(99.28)	0.07 (0.047,0.111)	0.07(0.046,0.11)	0.071(0.049,0.111)	0.67	0.858
PFBS	0.002	688(99.28)	0.038(0.029,0.147)	0.036(0.028,0.145)	0.04(0.03,0.149)	0.033	0.111
PFTrDA	0.0054	686(98.99)	0.381(0.31,0.495)	0.414(0.314,0.509)	0.366(0.308,0.442)	<0.001	0.002
PFUnDA	0.0022	683(98.56)	0.57(0.337,0.955)	0.589(0.345,0.994)	0.525(0.335,0.877)	0.156	0.389
PFDA	0.0014	678(97.84)	0.508(0.286,0.876)	0.522(0.315,0.879)	0.493(0.279,0.874)	0.423	0.705
PFBA	0.0034	445(64.21)	0.0108(<LOD,0.030)	0.0108(<LOD,0.0298)	0.012(<LOD,0.0312)	–	–
PFDoA	0.002	65(9.38)	<LOD(<LOD, <LOD)	<LOD(<LOD, <LOD)	<LOD(<LOD, <LOD)	–	–
PFTeDA	0.164	58(8.37)	<LOD(<LOD, <LOD)	<LOD(<LOD, <LOD)	<LOD(<LOD, <LOD)	–	–
PFHxA	0.0018	53(7.65)	<LOD(<LOD, <LOD)	<LOD(<LOD, <LOD)	<LOD(<LOD, <LOD)	–	–
PFHxDA	0.34	32(4.62)	<LOD(<LOD, <LOD)	<LOD(<LOD, <LOD)	<LOD(<LOD, <LOD)	–	–
PFDS	0.0138	5(0.72)	<LOD(<LOD, <LOD)	<LOD(<LOD, <LOD)	<LOD(<LOD, <LOD)	–	–
PFPeA	0.0022	4(0.58)	<LOD(<LOD, <LOD)	<LOD(<LOD, <LOD)	<LOD(<LOD, <LOD)	–	–
PFODA	0.6	2(0.29)	<LOD(<LOD, <LOD)	<LOD(<LOD, <LOD)	<LOD(<LOD, <LOD)	–	–

LOD, limit of detection for PFASs; FDR, false discovery rate. Detection ratios of PFBA, PFHxA, PFDoA, PFTeDA, PFHxDA, PFODA, PFDS and PFPeA were all < 70%; therefore, these values were not calculated. *P*-values were derived from the Mann–Whitney *U* test between the two groups.

The Spearman correlations between the 10 PFAS are displayed in Supplementary Figure S3. Significant positive correlations were observed among the 10 PFAS (*p* < 0.001). The highest correlation coefficient was observed between PFNA and PFDA [*ρ* (rho) = 0.83], followed by PFUnDA–PFDA (*ρ* = 0.82) and PFNA–PFUnDA (*ρ* = 0.78). Similarly, PFOS exhibited strong positive correlations with PFNA (*ρ* = 0.74), PFHpS (*ρ* = 0.76), PFUnDA (*ρ* = 0.78), and PFDA (*ρ* = 0.76).

### 3.3 Association of individual PFAS exposure with the odds of co-occurrence of GDM and PE/E

**Figure 1:**
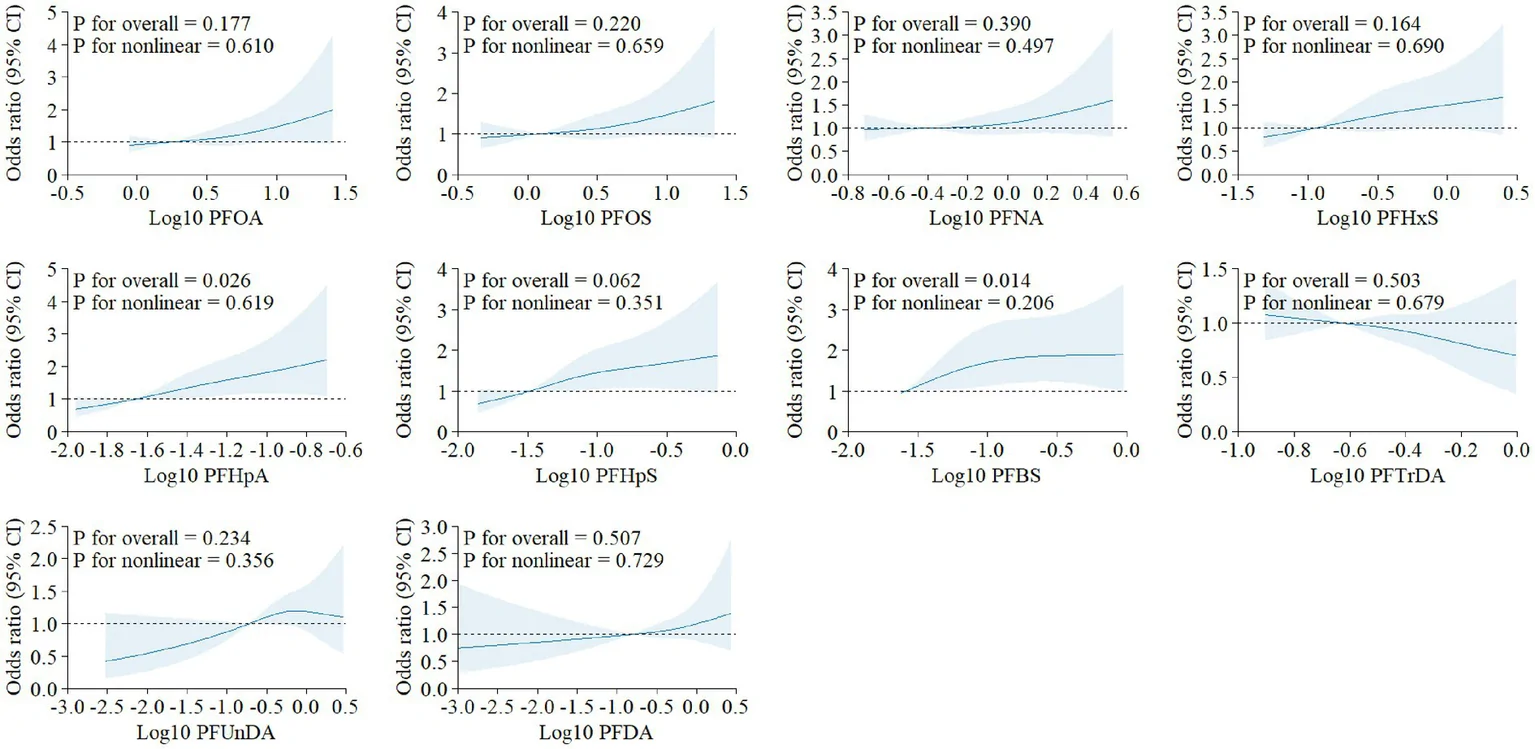
Restricted cubic splines for the relationship between the log10-transformed concentrations of PFAS and the co-occurrence of GDM and preeclampsia/eclampsia. Solid lines (blue) correspond to the odds ratios (ORs), and shaded regions correspond to the 95% confidence intervals (CIs) of the *beta* coefficient. Knots were fixed at the 10th, 50th, and 90th percentiles of the log10-transformed PFAS. The models were adjusted for maternal age (continuous variable), ppBMI (continuous variable), maternal ethnicity, maternal education level, method of conception, parental tobacco smoke exposure, maternal alcohol consumption, gravidity, parity and enrollment season.

The dose–response relationships between each of the 10 PFAS and the odds of co-occurrence of GDM and PE/E are presented in Figure 1. Significant overall associations with the co-occurrence odds were observed for PFHpA (*P*_overall_ = 0.026) and PFBS (*P*_overall_ = 0.014), and neither relationship exhibited a nonlinear pattern (*P*_non-linear_ = 0.619 for PFHpA; *P*_non-linear_ = 0.206 for PFBS). However, the overall associations were no longer statistically significant after FDR correction. No significant relationships were found for the remaining PFAS. The FDR *p*-values are provided in Supplementary Table S2.

The associations of individual PFAS with the odds of co-occurrence of GDM and PE/E from the multivariable logistic regression models are illustrated in Table 3. After FDR correction, each log10-unit increment in PFHpA and PFBS concentrations was significantly associated with increased odds of co-occurrence, with aORs of 2.439 (95% CI: 1.269–4.689) and 1.688 (95% CI: 1.161–2.453), respectively. For PFHpS, a positive association was also observed (aOR = 1.712, 95% CI: 1.051–2.788); however, the association was not statistically significant after FDR correction. The remaining seven PFAS showed no significant association with the odds of co-occurrence.

**Table 3:** Association between individual PFAS and the co-occurrence of GDM and PE/E.

PFAS concentrations (ng/mL)	cOR (95% CI)	*P*-value for trend	FDR *P*-value for trend	aOR (95% CI)^a^	*P*-value for trend	FDR *P*-value for trend
Log10 PFOA	1.186 (0.788–1.785)	0.414	0.689	1.642(0.944–2.858)	0.079	0.154
Log10 PFOS	1.126(0.766–1.654)	0.546	0.745	1.494 (0.936–2.383)	0.092	0.154
Log10 PFNA	1.133(0.714–1.799)	0.596	0.745	1.406(0.799–2.475)	0.237	0.263
Log10 PFHxS	1.056(0.877–1.272)	0.214	0.536	1.537(0.988–2.39)	0.056	0.141
Log10 PFHpA	2.303(1.344–3.946)	0.002	0.024	2.439(1.269–4.689)	0.008	0.038
Log10 PFHpS	1.085(0.931–1.265)	0.331	0.661	1.712(1.051–2.788)	0.031	0.102
Log10 PFBS	1.34(0.989–1.816)	0.059	0.197	1.688(1.161–2.453)	0.006	0.038
Log10 PFTrDA	0.548(0.295–1.016)	0.056	0.197	0.673(0.342–1.327)	0.253	0.263
Log10 PFUnDA	0.959(0.696–1.322)	0.8	0.889	1.346(0.912–1.988)	0.135	0.193
Log10 PFDA	1.092(0.868–1.374)	0.923	0.923	1.203(0.87–1.663)	0.263	0.263

GDM, gestational diabetes mellitus; PE/E, preeclampsia/eclampsia; cOR, crude odds ratio; aOR, adjusted odds ratio; CI, confidence interval.

^a^The models were adjusted for maternal age (continuous variable), ppBMI (continuous variable), maternal ethnicity, maternal education level, method of conception, parental tobacco smoke exposure, maternal alcohol consumption, gravidity, parity and enrollment season.

### 3.4 PFAS individual and combined exposure with the co-occurrence of GDM and PE/E based on BKMR

The posterior inclusion probabilities (PIPs) for each PFAS are provided in Supplementary Table S1. PFHpA and PFBS yielded the two highest PIP values across all tested PFAS congeners (0.3516 and 0.3488, respectively), indicative of their moderate importance in the mixture model.

**Figure 2:**
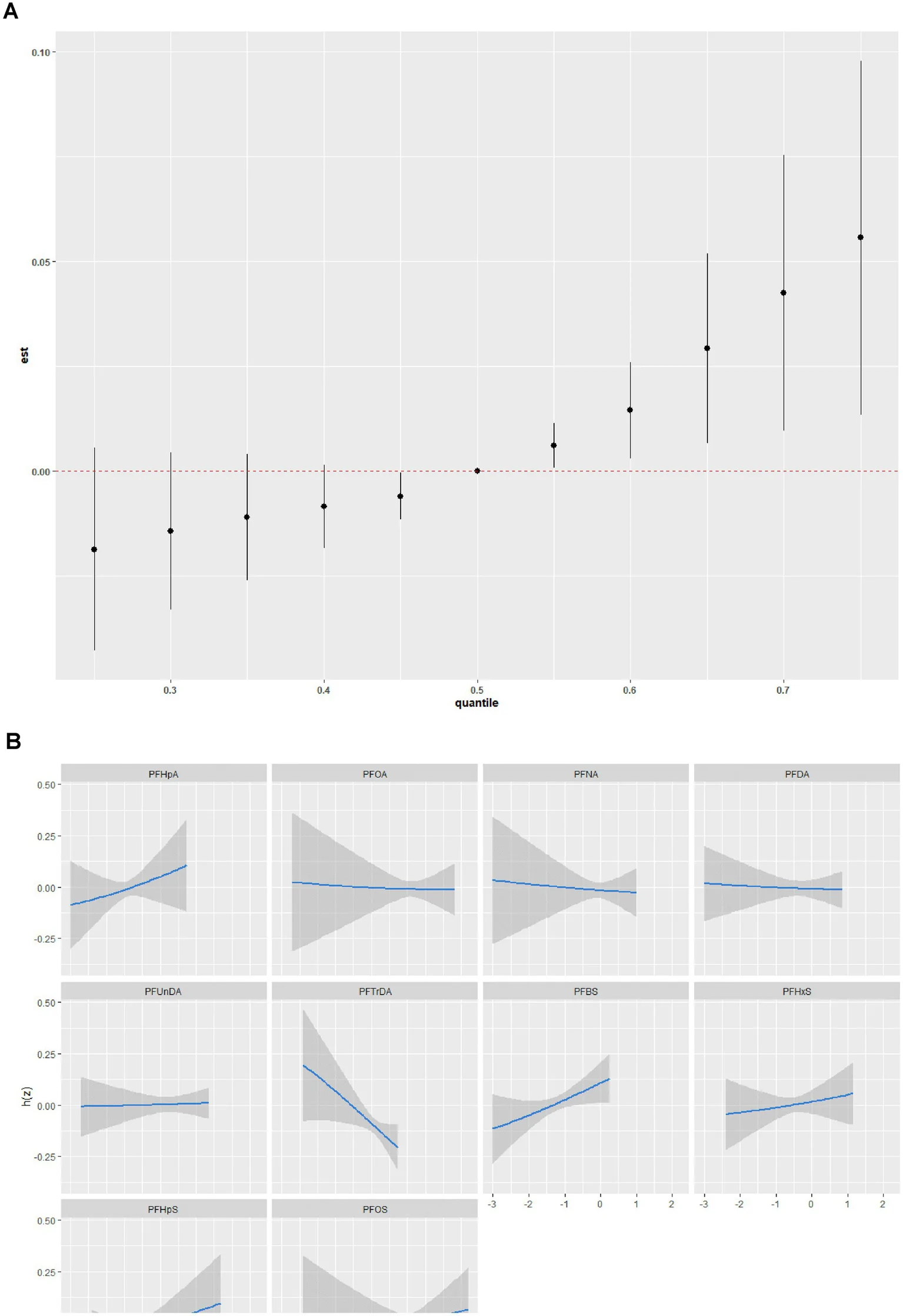
Bayesian kernel machine regression (BKMR) analysis. **(A)** Combined association of the 10 perfluoroalkyl substances (PFAS) with the co-occurrence of GDM and preeclampsia/eclampsia. All PFAS at particular percentiles (from 0.25 to 0.75 increment by 0.05) were compared to all the chemicals at their 50th percentile. **(B)** Univariate exposure–response functions and 95% confidence interval (CI) bands (gray area) for each PFAS, with other nine PFAS fixed at their median concentrations. The models were adjusted for maternal age (continuous variable), ppBMI (continuous variable), maternal ethnicity, maternal education level, method of conception, parental tobacco smoke exposure, maternal alcohol consumption, gravidity, parity and enrollment season.

The combined association of the PFAS mixture with the co-occurrence of GDM and PE/E are shown in Figure 2A. The association between the PFAS mixture and the co-occurrence showed an increasing trend. The odds of co-occurrence demonstrated a statistically significant increase when all the PFAS were fixed at their 55th percentile or above compared to their 50th percentile.

The exposure–response functions for each PFAS are presented in Figure 2B. When the other nine PFAS were fixed at their median levels, PFHpA, PFHpS, and PFBS were each positively and approximately linearly associated with the odds of co-occurrence of GDM and PE/E, whereas PFTrDA exhibited an approximately negative linear relationship.

Interactions between PFAS were assessed as presented in Supplementary Figure S4. Effect estimates for each individual PFAS remained broadly stable across quantiles of the remaining PFAS, indicating no robust evidence of pairwise PFAS interaction effects on the odds of co-occurrence of GDM and PE/E.

### 3.5 Sensitivity analyses among the participants with natural conception

Significant overall associations with co-occurrence odds of GDM and PE/E were observed for PFHpA (*P*_overall_ = 0.017) and PFBS (*P*_overall_ = 0.008), and neither relationship exhibited a nonlinear pattern (*P*_non-linear_ = 0.728 for PFHpA; *P*_non-linear_ = 0.224 for PFBS), as shown in Supplementary Figure S5. However, these associations did not survive after FDR correction. No significant relationships were found for the remaining PFAS. The FDR *p*-values are provided in Supplementary Table S4.

After FDR correction, each log10-unit increment in PFHpA and PFBS concentrations was significantly associated with increased co-occurrence odds, with aORs of 2.598 (95% CI: 1.343–5.024) and 1.768 (95% CI: 1.213–2.578), respectively. For PFHpS, a positive association was also observed (aOR = 1.742, 95% CI: 1.066–2.846); however, the association did not remain significant after FDR correction. The remaining seven PFAS showed no significant associations (Supplementary Table S5).

The combined association of the PFAS mixture with the co-occurrence of GDM and PE/E is shown in Supplementary Figure S6, which demonstrated an increasing trend, and the co-occurrence odds showed a statistically significant increase when all the PFAS were fixed at their 55th percentile or above compared to their 50th percentile.

### 3.6 Subgroup analyses stratified by maternal age

In the subgroup of mothers aged <35 years, no significant overall associations (all *P*_overall_ > 0.05) and no evident non-linear dose–response patterns (all *P*_non-linear_ > 0.05) were identified for any of the 10 PFAS, either before or after FDR correction (Supplementary Figure S7 and Supplementary Table S6). Each log10-unit increment in PFHpA and PFBS concentrations was significantly associated with increased co-occurrence odds, with aORs of 2.555 (95% CI: 1.114–5.859) and 1.694 (95% CI: 1.088–2.636), respectively; however, these associations did not remain significant after FDR correction. The remaining eight PFAS showed no significant associations (Supplementary Table S7). The combined association of the PFAS mixture with the co-occurrence of GDM and PE/E is shown in Supplementary Figure S8, which demonstrated an increasing trend, and the co-occurrence odds showed a statistically significant increase when all the PFAS were fixed at their 60th percentile or above compared to their 50th percentile.

In the subgroup of mothers aged ≥ 35 years, a significant overall association with co-occurrence odds was observed only for PFHpS (*P*_overall_ = 0.034), with no non-linear pattern (*P*_non-linear_ = 0.192) (Supplementary Figure S7). Each log10-unit increment in PFHpS was significantly associated with an increased co-occurrence odds (aOR = 2.947, 95% CI: 1.222–7.108). However, these associations did not remain significant after FDR correction. No significant associations were observed for the remaining nine PFAS (Supplementary Tables S6, S7). Furthermore, no significant combined association was found between PFAS mixture and the co-occurrence odds (Supplementary Figure S8).

## 4 Discussion

In the current study, multivariable logistic regression models revealed an association between maternal first-trimester exposure to PFBS or PFHpA and increased co-occurrence odds of GDM and PE/E, findings that were robust in sensitivity analyses. To our knowledge, direct evidence regarding these two PFAS subtypes and this specific co-occurrence is scarce. Thus, we compared our findings with existing studies on single pregnancy complications to interpret our results. For PFBS, our co-occurrence finding is partially aligned with prior single-disease evidence from the Chinese population-based cohorts which showed that elevated early-pregnancy PFBS levels were associated with an increased risk of GDM (26, 27). Similarly, higher PFBS concentrations were associated with increased odds of preeclampsia (32) and HDP (35). However, several studies reported no association between PFBS and GDM (28) or preeclampsia (38, 47). For PFHpA, the pattern is more equivocal. While our co-occurrence odds estimate was positive, previous single-disease studies have yielded mixed results. A Chinese study reported positive associations with GDM (27), but others found no association (28) or even protective effects (48). Similarly, for preeclampsia, a large cohort study from China and Sweden reported null associations for PFHpA (38, 47), whereas one study suggested a negative association (40).

Of particular interest, despite the substantial plasma levels of PFOA and PFOS observed in our cohort, no statistically significant association was found between these compounds and the odds of co-occurrence of GDM and PE/E. Our null findings for this co-occurrence are partially consistent with prior single-disease evidence. For GDM, null associations with PFOA or PFOS were reported in three Chinese cohorts (26, 27, 30, 49). For preeclampsia, null findings were similarly observed across Canadian, and Chinese studies (32, 33, 38, 50). A Swedish nationwide study further found inverse associations with GDM for both compounds, but no associations with preeclampsia (29). However, positive evidence also exists. For GDM, significant positive associations with PFOA or PFOS exposure were reported in a U. S. prospective cohort study (51) and in two Chinese case–control studies, which reported PFOA in one study (28), and PFOA and PFOS in the other (48). For PE/E, PFOS was positively associated with the risk of preeclampsia in American cohort studies (34), further supported by a Mendelian randomization study (36). Similarly, a Chinese nested case–control study reported that PFOS was positively associated with HDP (35). Additionally, evidence from a study in China suggested a positive association between PFOA exposure and preeclampsia (40).

Interestingly, contrary to our initial hypothesis, the co-occurrence group exhibited significantly reduced PFTrDA concentrations compared to controls, suggesting an inverse association. However, this finding was not corroborated by the RCS model, which showed no apparent association, nor by logistic regression analyses that treated PFTrDA as a continuous variable. Intriguingly, the exposure–response function in the BKMR model demonstrated an inverse association between PFTrDA levels and the odds of co-occurrence. The discrepancy across analytical methods may imply a complex, non-monotonic relationship between PFTrDA and the co-occurrence, or potential interactions with other co-exposures, which warrants further investigation in future studies. For PFHpS, no intergroup disparity was detected, and no clear association was detected in the RCS model. However, when analyzed as a continuous variable in logistic regression analyses, PFHpS showed a significant positive association with the odds of co-occurrence without adjustment for multiple comparisons; however, after FDR correction, the significance was no longer observed. In the BKMR model, the exposure-response function demonstrated increased odds of co-occurrence with rising PFHpS concentrations. Therefore, the significant findings for PFHpS should therefore be interpreted with caution.

In the present study, BKMR models revealed a positive association of PFAS exposure on the odds of co-occurrence of GDM and PE/E, with PFHpA and PFBS yielded the two highest PIP values across all tested PFAS congeners. This combined association for the co-occurrence partially aligns with prior single-disease evidence. For instance, consistent evidence from Chinese populations has indicated that exposure to PFAS mixtures was associated with an increased risk of GDM. However, the specific contributors varied across studies: a birth cohort study identified PFHpA as the largest contributor (27), whereas a case–control study highlighted PFOA as the main driver (48). Similarly, research on HDP has yielded comparable findings. A study in China reported a statistically significant elevation in HDP risk when all PFAS congeners were simultaneously increased from the 50th to the 60th percentile (35). Moreover, another study in China observed a rising trend in preeclampsia risk with increasing mixture concentrations, with PFOA emerging as the primary driver (40). However, our findings diverged from two prior studies, which documented that PFAS mixture exposure was not associated with the risk of GDM (28, 52). It should be noted that the combined association analysis of PFAS based on the equal-percentile assumption in BKMR is intended to avoid bias from subjective exposure-combination selection, rather than to imply equivalence in the relative toxicity of different congeners. Therefore, the interpretation should focus on the overall mixture trend rather than on the absolute effect estimates of individual substances.

The mechanisms underlying the association of maternal PFAS exposure with the co-occurrence of GDM and PE/E remain incompletely understood. Several biological pathways have been proposed to link PFAS exposure with GDM and PE/E, including placental dysfunction, oxidative stress, and inflammatory responses. As a principal target of PFAS bioaccumulation, the placenta exhibits marked susceptibility to these compounds, manifesting as aberrant weight, structural disorganization, and microthrombi. Placental insufficiency—characterized by low IGFBP1 expression linked to reduced insulin sensitivity, and inadequate trophoblast invasion with impaired spiral artery remodeling—may contribute to the pathogenesis of both GDM and PE/E (22, 23). Beyond placental damage, PFAS may also promote systemic inflammation and oxidative stress (24, 25). Accumulating evidence indicates that elevated proinflammatory cytokines exacerbate insulin resistance and hyperglycemia, while oxidative stress and lipid dyshomeostasis serve as early predictors of preeclampsia. Collectively, PFAS may contribute to the GDM–PE/E comorbidity through parallel pathways: direct placental impairment and systemic metabolic disruption driven by inflammation and oxidative stress. Given the observational nature of our study and the absence of mechanistic biomarkers, the biological pathways proposed above should be interpreted as speculative and hypothesis-generating, requiring confirmation in future experimental or longitudinal studies.

This study has several notable strengths. First, this is the first study to investigate the association of maternal PFAS exposure with the co-occurrence of GDM and PE/E. Second, our research is conducted within a prospective cohort, enabling the longitudinal collection of exposure and outcome data, thereby reducing the risk of selection and recall bias. Third, this study evaluates both individual and combined associations of PFAS with this co-occurrence, giving a clearer understanding of their association. Finally, participants were recruited from multiple provinces and diverse regions across China. This broader geographic and demographic coverage enhances the applicability of our results to more diverse populations. Nevertheless, certain limitations should be acknowledged. First, the assessment of plasma PFAS levels relies on a single measurement taken in early pregnancy. As previous studies have indicated that PFAS concentrations may be diluted by the increase in plasma volume during pregnancy; therefore, repeated measurements across pregnancy would better characterize the exposure. Second, in the co-occurrence of GDM and PE/E, GDM was diagnosed primarily based on the OGTT conducted during the second trimester. Therefore, incident cases of GDM developing later in pregnancy would have been missed, which may have attenuated the observed association between plasma PFAS levels and GDM risk. Third, we lacked data on serum creatinine or eGFR to adjust for potential renal clearance differences. While we excluded women with pre-existing kidney disease and restricted blood sampling to a narrow early-pregnancy window (12–14 weeks), we acknowledge that residual confounding by renal function cannot be excluded. Finally, the case group comprised only women with concurrent GDM and PE/E, without separate GDM-only or PE/E-only comparison groups. Consequently, we are unable to distinguish whether the observed associations are driven by GDM, by PE/E, or by their co-occurrence. Our findings should therefore be interpreted as exploratory evidence linking PFAS exposure to the comorbid state of these two conditions, rather than establishing disease-specific or co-occurrence-specific effects. Future studies with separate GDM-only and PE/E-only groups are warranted to further clarify this question.

In conclusion, our results revealed that PFHpA, PFBS, and the 10 PFAS mixture were all positively associated with increased odds of co-occurrence of GDM and PE/E.

## Data Availability

The raw data supporting the conclusions of this article will be made available by the authors, without undue reservation.

## Glossary

PFAS: per- and polyfluoroalkyl substances
GDM: gestational diabetes mellitus
PE/E: preeclampsia/eclampsia
UPLC-MS/MS: ultra-high performance liquid chromatography-tandem triple quadrupole mass spectrometry
RCS: restricted cubic spline
BKMR: Bayesian kernel machine regression
aOR: adjusted odds ratio
HDP: hypertensive disorders in pregnancy
FDR: false discovery rate
SGA: small-for-gestational-age
LGA: large-for-gestational-age
AFFF: aqueous film-forming foam
DEBC: drug exposure birth cohort
OGTT: oral glucose tolerance
IADPSG: International Association of Diabetes and Pregnancy Study Groups
FBG: fasting blood glucose
SBP: systolic blood pressure
DBP: diastolic blood pressure
PFHxS: perfluorohexane sulfonic acid
PFHpS: perfluoroheptane sulfonic acid
PFOS: perfluorooctane sulfonic acid
PFDS: perfluorodecane sulfonic acid
PFHpA: perfluoroheptanoic acid
PFOA: perfluorooctanoic acid
PFNA: perfluorononanoic acid
PFDA: perfluorodecanoic acid
PFUnDA: perfluoroundecanoic acid
PFDoA: perfluorododecanoic acid
PFTrDA: perfluorotridecanoic acid
PFTeDA: perfluorotetradecanoic acid
PFBA: perfluorobutanoic acid
PFPeA: perfluoropentanoic acid
PFBS: perfluorobutanesulfonic acid
PFHxA: perfluorohexanoic acid
PFHxDA: perfluorohexadecanoic acid
PFODA: perfluorooctadecanoic acid
LOD: limit of detection
ETS: environmental tobacco smoke
SD: standard deviation
IQR: interquartile ranges
CI: confidence interval
PIP: posterior inclusion probability
