# Supplementary material for: Maternal per- and polyfluoroalkyl substances exposure and the co-occurrence of gestational diabetes mellitus and preeclampsia/eclampsia: a nested case–control study
Source: Front Public Health. 2026 Sep 3;14:1929511. doi: 10.3389/fpubh.2026.1929511 (PMC13582519; doi:10.3389/fpubh.2026.1929511)
Supplement: Supplementary file 1 [file Data_sheet_1.docx]

Figure S1. The flowchart of participant selection.

Figure S2.Directed acyclic graph (DAG) for association between PFAS and the co-occurrence of gestational diabetes mellitus (GDM) and preeclampsia/eclampsia. Gray boxes = exposure and outcome; white boxes =potential confounders.

Figure S3. Spearman’s rank correlation coefficients of PFASs measured in maternal plasma samples.

Notes: The significance levels are set as *P* < 0.001 unless otherwise indicated (*, *P* < 0.05; #, non-significance). Gradient Color from red to blue represent the correlation coefficient (r).

Figure S4.Bivariate exposure–response functions between each pair of PFAS on the co-occurrence of gestational diabetes mellitus (GDM) and preeclampsia/eclampsia, with all other exposures fixed at their 50th percentile. The models were adjusted for maternal age (continuous variable), ppBMI (continuous variable), maternal ethnicity, maternal education level, method of conception, parental smoking or ETS, maternal alcohol consumption, gravidity, parity and enrollment season.

Figure S5. Restricted cubic splines for the relationship between the log10-transformed concentrations of PFAS and the co-occurrence of GDM and preeclampsia/eclampsia among the participants with natural conception. Solid lines (blue) correspond to the odds ratios (ORs), and shaded regions correspond to the 95% confidence intervals (CIs) of the beta coefficient. Knots were fixed at the 10th, 50th, and 90th percentiles of the log10-transformed PFAS. The models were adjusted for maternal age (continuous variable), ppBMI (continuous variable), maternal ethnicity, maternal education level, method of conception, parental tobacco smoke exposure, maternal alcohol consumption, gravidity, parity and enrollment season.

Figure S6. Combined association of the ten perfluoroalkyl substances (PFAS) with the co-occurrence of GDM and preeclampsia/eclampsia among the participants with natural conception by Bayesian kernel machine regression (BKMR) analysis. All PFAS at particular percentiles (from 0.25 to 0.75 increment by 0.05) were compared to all the chemicals at their 50th percentile. The models were adjusted for maternal age (continuous variable), ppBMI (continuous variable), maternal ethnicity, maternal education level, method of conception, parental tobacco smoke exposure, maternal alcohol consumption, gravidity, parity and enrollment season.

Figure S7. Restricted cubic splines for the relationship between the log10-transformed concentrations of PFAS and the co-occurrence of GDM and preeclampsia/eclampsia stratified by maternal age (A: < 35 years, B: ≥ 35 years). Solid lines (blue) correspond to the odds ratios (ORs), and shaded regions correspond to the 95% confidence intervals (CIs) of the beta coefficient. Knots were fixed at the 10th, 50th, and 90th percentiles of the log10-transformed PFAS. The models were adjusted for maternal age (continuous variable), ppBMI (continuous variable), maternal ethnicity, maternal education level, method of conception, parental tobacco smoke exposure, maternal alcohol consumption, gravidity, parity and enrollment season.

Figure S8. Bayesian kernel machine regression (BKMR) analysis. (A): Combined association of the ten perfluoroalkyl substances (PFAS) with the co-occurrence of GDM and preeclampsia/eclampsia stratified by maternal age (A: < 35 years, B: ≥ 35 years). All PFAS at particular percentiles (from 0.25 to 0.75 increment by 0.05) were compared to all the chemicals at their 50th percentile. The models were adjusted for maternal age (continuous variable), ppBMI (continuous variable), maternal ethnicity, maternal education level, method of conception, parental tobacco smoke exposure, maternal alcohol consumption, gravidity, parity and enrollment season.

**Table S1. Covariates definitions and categorization**

| **Variable** | **Definition/Categorization** |
| --- | --- |
| Maternal age | Continuous (at the time of the last menstrual period, years) |
| Pre-pregnancy BMI | Continuous (kg/m²) |
| Ethnicity | Categorical (Han, or others) |
| Education level | Ordinal (junior high or lower, high school, college or higher) |
| Method of conception | Categorical (natural conception, or medically assisted reproduction) |
| Parental tobacco smoke exposure | Binary (Yes or No, defined as either parent smoked or was exposed to secondhand smoke from 3 months before conception to the first trimester) |
| Maternal alcohol consumption | Binary (Yes or No, defined as any alcohol intake during the period from 3 months before pregnancy through the first trimester) |
| Gravidity | Binary (Primigravida or Multigravida) |
| Parity | Binary (Nulliparous or Parous) |
| Enrollment season | Categorical (Spring, Summer, Autumn, Winter) |

**Table S2. FDR corrected *P*-values in the RCS analysis**

| Exposure | Overall *P*-values | Overall FDR *P*-values | Nonlinear *P*-values | Nonlinear FDR *P*-values |
| --- | --- | --- | --- | --- |
| PFOA | 0.177 | 0.334 | 0.610 | 0.729 |
| PFOS | 0.220 | 0.334 | 0.659 | 0.729 |
| PFNA | 0.390 | 0.488 | 0.497 | 0.729 |
| PFHxS | 0.164 | 0.334 | 0.690 | 0.729 |
| PFHpA | 0.026 | 0.129 | 0.619 | 0.729 |
| PFHpS | 0.062 | 0.208 | 0.351 | 0.729 |
| PFBS | 0.014 | 0.129 | 0.206 | 0.729 |
| PFTrDA | 0.503 | 0.507 | 0.679 | 0.729 |
| PFUnDA | 0.234 | 0.334 | 0.356 | 0.729 |
| PFDA | 0.507 | 0.507 | 0.729 | 0.729 |

**Table S3. Posterior inclusion probabilities estimated by BKMR model.**

| PFAS | PIP^a^ |
| --- | --- |
| PFHpA | 0.3516 |
| PFBS | 0.3488 |
| PFTrDA | 0.2416 |
| PFHpS | 0.148 |
| PFOS | 0.1188 |
| PFHxS | 0.1024 |
| PFOA | 0.0956 |
| PFUnDA | 0.0412 |
| PFDA | 0.0328 |
| PFNA | 0.0164 |

a: Posterior inclusion probabilities for each PFAS in component-wise variable selection.

**Table S4 FDR corrected *P*-values in the RCS analysis among the participants with natural conception**

| Exposure | Overall *P*-values | Overall FDR *P*-values | Nonlinear *P*-values | Nonlinear FDR *P*-values |
| --- | --- | --- | --- | --- |
| PFOA | 0.108 | 0.255 | 0.544 | 0.748 |
| PFOS | 0.153 | 0.255 | 0.552 | 0.748 |
| PFNA | 0.305 | 0.381 | 0.447 | 0.748 |
| PFHxS | 0.131 | 0.255 | 0.748 | 0.748 |
| PFHpA | 0.017 | 0.085 | 0.728 | 0.748 |
| PFHpS | 0.053 | 0.177 | 0.327 | 0.748 |
| PFBS | 0.008 | 0.080 | 0.224 | 0.748 |
| PFTrDA | 0.543 | 0.543 | 0.730 | 0.748 |
| PFUnDA | 0.214 | 0.305 | 0.461 | 0.748 |
| PFDA | 0.402 | 0.446 | 0.615 | 0.748 |

**Table S5 Association between individual PFAS and the co-occurrence of GDM and PE/E among the participants with natural conception**

| PFAS concentrations (ng/mL) | | cOR (95% CI) | | *P*-value  for trend | | FDR *P*  -value  for trend | | aOR (95% CI)^a^ | *P*-value  for trend | | FDR *P*  -value  for trend | |  |
| --- | --- | --- | --- | --- | --- | --- | --- | --- | --- | --- | --- | --- | --- |
| log10 PFOA | 1.193(0.772-1.844) | | 0.427 | | 0.747 | | 1.762(1.003-3.096) | | | 0.049 | | 0.098 | |
| log10 PFOS | 1.076(0.722-1.603) | | 0.719 | | 0.842 | | 1.557(0.972-2.492) | | | 0.065 | | 0.109 | |
| log10 PFNA | 1.05(0.651-1.694) | | 0.842 | | 0.842 | | 1.472(0.833-2.602) | | | 0.184 | | 0.230 | |
| log10 PFHxS | 1.241(0.847-1.818) | | 0.267 | | 0.668 | | 1.586(1.018-2.472) | | | 0.041 | | 0.098 | |
| log10 PFHpA | 1.889(1.079-3.306) | | 0.026 | | 0.130 | | 2.598(1.343-5.024) | | | 0.005 | | 0.023 | |
| log10 PFHpS | 1.175(0.774-1.784) | | 0.448 | | 0.747 | | 1.742(1.066-2.846) | | | 0.027 | | 0.090 | |
| log10 PFBS | 1.443(1.048-1.987) | | 0.025 | | 0.130 | | 1.768(1.213-2.578) | | | 0.003 | | 0.023 | |
| log10 PFTrDA | 0.567(0.309-1.042) | | 0.068 | | 0.225 | | 0.683(0.346-1.348) | | | 0.271 | | 0.271 | |
| log10 PFUnDA | 0.918(0.661-1.277) | | 0.612 | | 0.842 | | 1.392(0.94-2.062) | | | 0.099 | | 0.141 | |
| log10 PFDA | 0.964(0.727-1.28) | | 0.802 | | 0.842 | | 1.233(0.889-1.709) | | | 0.209 | | 0.232 | |

Note: GDM, gestational diabetes mellitus; PE/E, preeclampsia/eclampsia; cOR, crude odds ratio; aOR, adjusted odds ratio; CI, confidence interval.

^a^ The models were adjusted for maternal age (continuous variable), ppBMI (continuous variable), maternal ethnicity, maternal education level, method of conception, parental tobacco smoke exposure, maternal alcohol consumption, gravidity, parity and enrollment season.

**Table S6 FDR corrected *P*-values in the RCS analysis stratified by maternal age**

| Exposure | Overall *P*-values | Overall FDR *P*-values | Nonlinear *P*-values | Nonlinear FDR *P*-values |
| --- | --- | --- | --- | --- |
| < 35 years |  |  |  |  |
| PFOA | 0.294 | 0.647 | 0.209 | 0.932 |
| PFOS | 0.497 | 0.647 | 0.598 | 0.932 |
| PFNA | 0.518 | 0.647 | 0.476 | 0.932 |
| PFHxS | 0.179 | 0.596 | 0.652 | 0.932 |
| PFHpA | 0.092 | 0.460 | 0.939 | 0.977 |
| PFHpS | 0.491 | 0.647 | 0.374 | 0.932 |
| PFBS | 0.054 | 0.460 | 0.598 | 0.932 |
| PFTrDA | 0.693 | 0.770 | 0.919 | 0.977 |
| PFUnDA | 0.410 | 0.647 | 0.422 | 0.932 |
| PFDA | 0.796 | 0.796 | 0.977 | 0.977 |
| ≥ 35 years |  |  |  |  |
| PFOA | 0.585 | 0.960 | 0.800 | 0.994 |
| PFOS | 0.699 | 0.960 | 0.685 | 0.994 |
| PFNA | 0.968 | 0.968 | 0.953 | 0.994 |
| PFHxS | 0.866 | 0.962 | 0.994 | 0.994 |
| PFHpA | 0.399 | 0.960 | 0.516 | 0.994 |
| PFHpS | 0.034 | 0.342 | 0.192 | 0.908 |
| PFBS | 0.407 | 0.960 | 0.272 | 0.908 |
| PFTrDA | 0.294 | 0.960 | 0.206 | 0.908 |
| PFUnDA | 0.674 | 0.960 | 0.542 | 0.994 |
| PFDA | 0.768 | 0.960 | 0.868 | 0.994 |

**Table S7 Association between individual PFAS and the co-occurrence of GDM and PE/E stratified by maternal age**

| PFAS concentrations (ng/mL) | | cOR (95% CI) | | *P*-value  for trend | | FDR *P*  -value  for trend | | aOR (95% CI)^a^ | *P*-value  for trend | | FDR *P*  -value  for trend | |  |
| --- | --- | --- | --- | --- | --- | --- | --- | --- | --- | --- | --- | --- | --- |
| < 35 years |  | |  | |  | |  | | |  | |  | |
| log10 PFOA | 1.227(0.738-2.041) | | 0.430 | | 0.717 | | 1.406(0.696-2.841) | | | 0.342 | | 0.439 | |
| log10 PFOS | 1.163(0.736-1.836) | | 0.518 | | 0.740 | | 1.375(0.777-2.434) | | | 0.274 | | 0.439 | |
| log10 PFNA | 1.307(0.752-2.274) | | 0.342 | | 0.685 | | 1.403(0.682-2.887) | | | 0.358 | | 0.439 | |
| log10 PFHxS | 1.339(0.872-2.057) | | 0.182 | | 0.455 | | 1.647(0.979-2.769) | | | 0.060 | | 0.200 | |
| log10 PFHpA | 2.201(1.147-4.225) | | 0.018 | | 0.177 | | 2.555(1.114-5.859) | | | 0.027 | | 0.134 | |
| log10 PFHpS | 1.114(0.682-1.819) | | 0.667 | | 0.833 | | 1.3(0.71-2.378) | | | 0.395 | | 0.439 | |
| log10 PFBS | 1.411(0.987-2.018) | | 0.059 | | 0.297 | | 1.694(1.088-2.636) | | | 0.020 | | 0.134 | |
| log10 PFTrDA | 0.635(0.326-1.236) | | 0.181 | | 0.455 | | 0.713(0.33-1.542) | | | 0.390 | | 0.439 | |
| log10 PFUnDA | 0.976(0.671-1.421) | | 0.901 | | 0.901 | | 1.305(0.812-2.097) | | | 0.272 | | 0.439 | |
| log10 PFDA | 0.977(0.721-1.322) | | 0.879 | | 0.901 | | 1.134(0.783-1.642) | | | 0.505 | | 0.505 | |
| ≥ 35 years |  | |  | |  | |  | | |  | |  | |
| log10 PFOA | 1.133(0.55-2.334) | | 0.735 | | 0.928 | | 1.732(0.626-4.794) | | | 0.291 | | 0.534 | |
| log10 PFOS | 1.038(0.505-2.134) | | 0.918 | | 0.928 | | 1.503(0.627-3.602) | | | 0.361 | | 0.534 | |
| log10 PFNA | 0.798(0.334-1.907) | | 0.612 | | 0.928 | | 1.222(0.452-3.301) | | | 0.693 | | 0.693 | |
| log10 PFHxS | 1.067(0.512-2.222) | | 0.862 | | 0.928 | | 1.37(0.557-3.37) | | | 0.493 | | 0.548 | |
| log10 PFHpA | 2.545(0.97-6.681) | | 0.058 | | 0.540 | | 1.977(0.65-6.012) | | | 0.230 | | 0.534 | |
| log10 PFHpS | 1.451(0.725-2.902) | | 0.293 | | 0.928 | | 2.947(1.222-7.108) | | | 0.016 | | 0.161 | |
| log10 PFBS | 1.179(0.659-2.11) | | 0.580 | | 0.928 | | 1.472(0.696-3.112) | | | 0.311 | | 0.534 | |
| log10 PFTrDA | 0.269(0.054-1.334) | | 0.108 | | 0.540 | | 0.412(0.075-2.281) | | | 0.310 | | 0.534 | |
| log10 PFUnDA | 0.915(0.495-1.691) | | 0.776 | | 0.928 | | 1.343(0.639-2.821) | | | 0.436 | | 0.545 | |
| log10 PFDA | 1.029(0.551-1.921) | | 0.928 | | 0.928 | | 1.4(0.667-2.941) | | | 0.374 | | 0.534 | |

Note: GDM, gestational diabetes mellitus; PE/E, preeclampsia/eclampsia; cOR, crude odds ratio; aOR, adjusted odds ratio; CI, confidence interval.

^a^ The models were adjusted for maternal age (continuous variable), ppBMI (continuous variable), maternal ethnicity, maternal education level, method of conception, parental tobacco smoke exposure, maternal alcohol consumption, gravidity, parity and enrollment season.
